# Induced seismicity in coupled subsurface systems

**DOI:** 10.1098/rsta.2023.0193

**Published:** 2024-08-09

**Authors:** Adriana Paluszny, Ryan Schultz, Günter Zimmermann

**Affiliations:** ^1^ Imperial College London, London, UK; ^2^ Swiss Seismological Service, ETH Zürich, Zürich, Switzerland; ^3^ Helmholtz Centre Potsdam GFZ German Research Centre For Geosciences, Potsdam, Germany

**Keywords:** seismicity, induced seismicity, earthquakes, geomechanics, seismology

Induced seismicity is the anthropogenic generation of tremors and seismic events that locally alter the state of subsurface stresses. The occurrence of induced seismicity during the deployment of subsurface technologies can cause projects to be arrested, as was the recent case in the United Kingdom during shale gas development and has been the case in many other locations worldwide, affecting the development of important green transition and energy security technologies that are being developed.

Subsurface renewable energy technologies, such as carbon storage, geothermal energy extraction, hydrogen storage and compressed-air energy storage, are at the heart of the large-scale decarbonization of society and the sustainable development of an energy-secure future. All these technologies, which interact with the subsurface, have the potential to induce earthquakes, affecting the societal acceptance and financial viability of these projects at a large scale. There is currently a worldwide race to understand the controlling mechanisms of induced seismicity and how it depends not only on injection parameters and *in situ* stresses, but how it is also affected by rock and fluid properties and shifts to these properties, owing to natural spatial variations and temporally evolving geomechanical changes to the properties of the pre-existing discontinuities such as fractures and faults.

Subsurface operations, such as carbon sequestration, can leverage the pore space in rock formations buried at depth to provide final containment of fluids over thousands of years and at large scale. The variability of the subsurface also supports the storage of fluids over short periods of time (months to years), such as in the case of hydrogen, compressed air storage and thermal energy storage, which have the potential to store surplus renewable energy seasonally, to balance consumption and production rates of renewable energy throughout the year. These technologies rely on the injection, storage and withdrawal of fluids into the subsurface and vary greatly across temporal and spatial scales. However, injection into the subsurface is known to perturb the mechanical state of equilibrium of faults, which can lead to slip, potentially causing induced seismicity. This seismicity can be felt at the surface and is largely dependent on the magnitude of the displacement, the depth of the event and the characteristics of the rock and soil that lie over the fault that has been reactivated.

Induced seismicity has dramatically increased over the past 10 years, owing to the extensive hydraulic fracturing operations conducted worldwide, contributing to the negative social perception of the industry and leading to distinct regulatory dichotomies in the manner of how induced seismicity is managed. For example, some induced seismicity cases have caused economic/human losses through ground-shaking hazards; in other extreme cases, moratoriums on resource development have been emplaced owing to social concerns about these risks. Thus, there is a need to effectively manage these risks to avoid either type of loss. Even though there have been conserted efforts to minimize and prevent such occurrences in a systematic manner, induced seismicity is still a prevalent subsurface response to injection throughout the world, leading also to increased financial risk of subsurface technologies vital to large-scale decarbonization and sustainable energy storage. For many developing green technologies, proper management of induced seismicity will be a critical issue towards influencing public perception and promoting wide adoption.

In the context of the green transition, rock properties, fluid density, cyclic injection and spatial *in situ* heterogeneities have become important *foci* of research exploring the fundamental controlling mechanisms of induced seismicity. There are ongoing efforts to understand the hydromechanical factors that affect induced seismicity, with the objective of developing new effective strategies to minimize the effects felt on the surface and to de-risk future subsurface operations. This issue aims to capture the state-of-the-art and novel research in this area, as of early 2024, to provide important lessons learned to directly support challenges faced by green-transition technologies.

This issue presents a comprehensive exploration of fluid-induced seismicity across various energy production and storage technologies. The articles used a diverse range of methodologies, including numerical simulations, laboratory experiments, statistical analysis and seismic monitoring, to investigate induced seismicity. Across the papers, there is a shared focus on understanding the interplay between fluid injection, fault behaviour and subsurface structures. These diverse approaches enable a comprehensive exploration of fluid-induced seismicity across various geological settings and energy production technologies and contribute to our understanding of induced seismicity mechanisms, offering valuable insights for the mitigation of seismic hazards.

Esmaeilzadeh *et al*. [1] investigated fluid-induced seismicity associated with sealing faults in the Triassic Montney Formation in Western Canada. The study reveals a significant spatial correlation between induced seismicity and high lateral gradients in pore pressure, indicating the importance of sealing faults in understanding induced seismic risk. Analysis of hydraulic fracturing operations near a 4.5 M_L_ earthquake epicentre shows a large lateral pressure gradient, suggesting the potential for sealing faults to increase seismic hazard.

Zhang *et al.* [2] investigated fluid-induced seismicity in enhanced geothermal systems by comparing direct and indirect fluid injection into faults in granite rock samples. Results show that injecting fluid adjacent to a fault, as well as directly into it, can induce seismic hazards owing to high fluid pressure creating new fractures. Identifying pre-existing faults is crucial to mitigate seismic risks during enhanced geothermal systems operations, requiring immediate action if faults are detected during hydraulic stimulation.

Dang-Trung *et al.* [3] combined a multi-point flux approximation of flow with a contact mechanics approach, using a dual-mesh discretization, to show fracture propagation in two dimensions during injection near a fault in the context of geothermal reservoirs.

Verdon *et al.* [4] investigated the dynamics of induced seismicity from long-term fluid injection across 20 case studies. Using the seismogenic index and seismic efficiency, they analysed seismicity rates against injection rates. Cumulative values steeply rise within one to three years of injection initiation, stabilizing thereafter. Time-windowed values peak within 25–35% of the sequence, then decline. This pattern reflects early high pore pressure changes diminishing over time. Models based on the seismogenic index and seismic efficiency show significant correlations between observed and predicted magnitudes, providing scientific evidence that understanding seismicity rate variations aids in calibrating site-specific pore pressure models for more accurate hazard forecasting and mitigation strategies.

Boyet *et al.* [5] studied induced seismicity challenges in enhanced geothermal systems by combining a hydro-mechanical model with a seismicity rate model, to forecast mainshocks and aftershocks induced by fluid injection. Analysing Basel enhanced geothermal systems data, constant injection emerges as the most efficient strategy, enhancing fault permeability with limited post-injection seismicity. The hybrid model offers a versatile approach adaptable to various injection protocols, aiding safe enhanced geothermal system development.

Schultz’s [6] study presents a suite of statistical tools to infer maximum earthquake magnitudes from seismic catalogues. Through hypothesis testing, maximum likelihood estimation and ensemble weighting, these tools analyse induced seismicity data from various sources, revealing no evidence of volume-based influences restricting earthquake magnitude growth. Instead, an unbounded magnitude distribution adequately explains all cases tested. This suggests that induced earthquake hazards should be treated as unbounded, affecting hazard mitigation strategies. The developed tools offer a crucial means of understanding earthquakes and managing associated risks, with potential implications for future hazard mitigation efforts.

Langenbruch’s [7] study investigates the factors influencing maximum magnitudes of induced earthquakes, focusing on pressure diffusion in the Earth’s crust. Analysing global energy project data, they correlated maximum magnitudes with pressure diffusion length, noting increasing nucleation potential over time. The nucleation potential for larger earthquakes increases over time owing to diffusion-controlled fault growth. Their model aligns with observations and suggests maximum magnitudes can surpass expectations based on fluid volume alone. The work provides evidence that the identification of larger-scale, pre-existing and critically stressed faults is instrumental to understand and mitigate induced seismic hazards.

Dunham [8] extended models of fluid-driven fault slip by incorporating permeability enhancement and dilatancy effects that may occur during deformation. Dunham proposed that permeability enhancement and dilatancy, occurring instantaneously upon fault slip, can significantly influence the dynamics of fluid-driven shear fractures in low-permeability rocks, offering new insights into potential underlying mechanisms of fluid-driven fracture growth in understressed conditions.

Wang *et al.* [9] investigated seismic activity induced by fluid injection in the Raton Basin, focusing on the interaction between seismic sources and subsurface structures. Through seismic monitoring and receiver function analysis, active fault segments and spatiotemporal patterns are revealed. Complex fault clusters near injection wells contrast with simpler structures farther away, while abrupt structural transitions coincide with seismic activity. The research underscores the significance of structural heterogeneities in influencing induced seismicity, with findings suggesting that fluid connectivity between injection depths and basement faults plays a crucial role.

Burtonshaw *et al.* [10] investigated the effects of reservoir mechanical properties on induced seismicity during subsurface hydrogen storage. Through numerical simulations, this study assesses how variations in mechanical properties, such as Young’s modulus, Poisson’s ratio and Biot coefficient, affect fault slip in a porous depleted subsurface reservoir. The study finds that high Young’s modulus (greater than 40 GPa), Poisson’s ratio (greater than 0.30) and Biot coefficient (greater than 0.65) are preferable for minimizing seismic risk during low-density gas storage, such as hydrogen. Conversely, lower values of these properties increase the potential for induced seismic events at high injection rates, highlighting the need for careful selection of storage sites with suitable mechanical properties.

These findings collectively contribute to evidence-based policymaking and regulatory processes, promoting the development of large-scale renewable energy, energy storage and decarbonization technologies reliant on sustainable subsurface operations.

This issue is aimed at the wider academic community, regulators and government agencies seeking to support industry growth through evidence-based policies. It focuses on quantifying induced seismicity with physics-based evidence, which is required to maintain the framework of sustainable subsurface development. Our special issue is aimed at addressing key aspects such as financial investment risk, social perception and sustainable energy security. In particular, the UK government may need to consider adjustments to the traffic light system, which currently targets induced seismicity related to shale gas extraction. As green energy subsurface industries evolve, this system will probably require re-evaluation to accommodate the flexibility necessary for the development and refinement of storage mechanisms. This issue aims to highlight crucial new findings to better support this regulatory process, particularly in fostering the development of renewable energy and decarbonization technologies that depend on the subsurface for fluid storage, withdrawal or injection.

We hope the articles in this volume illustrate both the achievements and challenges in the dynamic, multidisciplinary field of induced seismicity management. Especially as this field undergoes an exciting and rapid evolution, ultimately contributing to sustainable energy solutions and the advancement of our civilization.

## Data Availability

This article has no additional data.
